# Effects and Mechanisms of Calcium Ion Addition on Lead Removal from Water by *Eichhornia crassipes*

**DOI:** 10.3390/ijerph17030928

**Published:** 2020-02-02

**Authors:** Jin-mei Zhou, Zhong-cheng Jiang, Xiao-qun Qin, Lian-kai Zhang, Qi-bo Huang, Guang-li Xu

**Affiliations:** 1 Institute of Karst Geology, Chinese Academy of Geological Sciences, Guilin 541004, China; 2 Faculty of Engineering, China University of Geosciences, Wuhan 430074, China; xu1963@cug.edu.cn; 3 Key Laboratory of Karst Ecosystem and Treatment of Rocky Desertification, Ministry of Natural Resources, Guilin 541004, China; 4 Key Laboratory of Karst Dynamics, Ministry of Natural Resources, Guilin 541004, China

**Keywords:** *Eichhornia crassipes*, lead, calcium ion (II), alleviation, cation exchange, functional groups

## Abstract

Karst water is rich in calcium ions (Ca^2+^) and exhibits poor metal availability and low biodegradation efficiency. This study sought to analyze the effects and mechanisms of Ca^2+^ on lead (Pb) removal and absorption by *Eichhornia crassipes* (a floating plant common in karst areas). Moreover, the morphology and functional groups of *E. crassipes* in water were characterized via SEM, and FTIR. The results demonstrated that the removal rate of Pb in karst water (85.31%) was higher than that in non-karst water (77.04%); however, the Pb bioconcentration amount (BCA) in *E. crassipes* roots in karst water (1763 mg/kg) was lower than that in non-karst water (2143 mg/kg). With increased Ca^2+^ concentrations (60, 80, and 100 mg/L) in karst water, the Pb removal rate increased (85.31%, 88.87%, and 92.44%), the Pb BCA decreased (1763, 1317, and 1095 mg/kg), and the Ca BCA increased (6801, 6955, and 9368 mg/kg), which was attributed to PbCO_3_ and PbSO_4_ precipitation and competitive Ca and Pb absorption. High Ca^2+^ concentrations increased the strength of cation exchange, alleviated the fracture degree of fibrous roots, reduced the atrophy of vascular bundles, protected the cell wall, promoted C–O combined with Pb, enhanced the strength of O‒H, SO_4_^2−^, C=O, and reduced the oxidization of alkynyl acetylene bonds.

## 1. Introduction

Karst water quality has begun to deteriorate in recent years due to increased industrial mining, agriculture, and other human activities. The “three wastes” generated by industrial and mining enterprises, municipal sewage discharge, and the use of heavy-metal-containing products have caused serious heavy metal environmental pollution [1]. Karst areas in China are mainly distributed in the southwestern region and are characterized by complex landforms and carbonate formations. Karst areas possess abundant groundwater resources, which are alkaline and rich in calcium ions (Ca^2+^) and exhibit poor metal availability and low biodegradation efficiency. Notably, karst water is characteristically rich in HCO_3_-Ca due to the dissolution of carbonate rocks [2]. Moreover, due to its unique hydrologic double-layer structure (i.e., surface and underground water), karst water is easily polluted but difficult to treat once it has been contaminated by heavy metals [3]. Heavy metal pollution in karst water has become an urgent, worldwide problem. Lead (Pb) is one of the most toxic heavy metal elements and has very long retention times, which is why it has become one of the most studied elements in the field of heavy metal pollution [4]. Aquatic Pb pollution mainly originates from industrial wastewater discharged by the lead smelting industry. Pb remains and accumulates in the food chain after entering the environment, which causes serious toxicity to organisms. Moreover, Pb affects plant photosynthesis and transpiration capacity [5,6]; therefore, Pb remediation has become the focus of many studies worldwide.

*Eichhornia crassipes* is an aquatic floating plant that is widely distributed all over the world and occurs in large quantities in karst areas in China. *E. crassipes* has broad, thick, glossy, ovate leaves and stems, which are important for photosynthesis, food production, gas exchange, and water transpiration [7]. This plant is known to adapt to a wide variety of environmental conditions and even grows well in sewage water. Importantly, *E. crassipes* possesses a high heavy metal tolerance and uptake efficiency and has therefore become popular for water phytoremediation [8]. *E.* *crassipes* can reproduce quickly and absorb various pollutants [9]. Suryandari et al. [10] also demonstrated Pb removal efficiencies of up to 99.71% in *E. crassipes* harvested after nine days.

Calcium (Ca) is an essential element for plants; it is an important regulator of plant growth and supports the structure and stability of cell walls, membranes, and membrane-binding proteins. Ca is also a second messenger that regulates the response of plants to environmental changes [11]. Moreover, certain Ca^2+^ concentrations could promote growth and development, metabolism, cell structure, and adaptability to heavy metal stress [12,13,14]. Ca^2+^ could also inhibit the transport of heavy metals from underground to aboveground plant structures [15]. Increased Ca^2+^ concentrations could reduce heavy metal solubility [16] and alleviate heavy metal phytotoxicity [17]. However, there is currently no consensus regarding the effects of Ca^2+^ on heavy metal accumulation in plants. Heeraman et al. [18] reported that high Ca^2+^ concentrations could promote the accumulation of arsenic in *Zorro fescue* (a grass species). In contrast, Calile et al. [16] demonstrated that Ca^2+^ had no apparent effect on the absorption of arsenic in the Chinese brake (*Pteris vittata*). Regardless of these discrepancies, high Ca^2+^ concentrations in karst water may be responsible for the higher heavy metal bioaccumulation capacity of some aquatic plants in karst waters than that in other environments [19]. However, the effects of Ca^2+^ on the Pb removal and accumulation capacity of *E. crassipes* in Pb-contaminated karst water remain unclear.

Therefore, this study sought to characterize the effect of Ca^2+^ on the Pb removal efficiency of *E. crassipes* in karst water. This study employed Inductively Coupled Plasma Mass Spectrometry (ICP-MS), Scanning Electron Microscopy (SEM), Fourier-Transform Infrared Spectrometry (FTIR), and other methods to investigate the Pb removal efficiency and absorption capacity of *E. crassipes* in karst and non-karst water at different Ca^2+^ concentrations. This study thus provides a comprehensive analysis of the mechanisms by which Ca^2+^ modulates Pb uptake by *E. crassipes* in karst water under Pb-induced stress, and provides a theoretical basis for the effective application of *E. crassipes* for Pb remediation in polluted karst water.

## 2. Materials and Methods

### 2.1. Materials

Similarly sized wild *E. crassipes* plants were collected from the Huixian wetland (Guilin, Guangxi, China), where these plants are abundant and widely distributed. After discarding decomposed leaves, the surface dirt on the remaining leaves was removed by repeatedly washing them with tap water and then with de-ionized water. The *E. crassipes* plants used herein had the following characteristics: weight, 150 g; height, 30 cm; Ca, 13,275 mg/kg; Mg, 2325 mg/kg; K, 28,950 mg/kg; Na, 1198 mg/kg; P, 2695 mg/kg; Fe, 2570 mg/kg; Mn, 598 mg/kg; Pb, 2.46 mg/kg; Cd, 0.118 mg/kg; Cu, 16.5 mg/kg; Zn, 53.6 mg/kg.

The karst and non-karst water used in the experiments were obtained from the outlets of the Mamian Shizishan underground river at Huixian and the Lijiang River in Guilin, respectively, using polyethylene barrels. Table 1 summarizes the karst and non-karst water hydrochemical parameters determined by this study. All chemicals used for experimental purposes were of analytical grade and were dissolved in de-ionized water.

### 2.2. Methods

**Figure 1:**
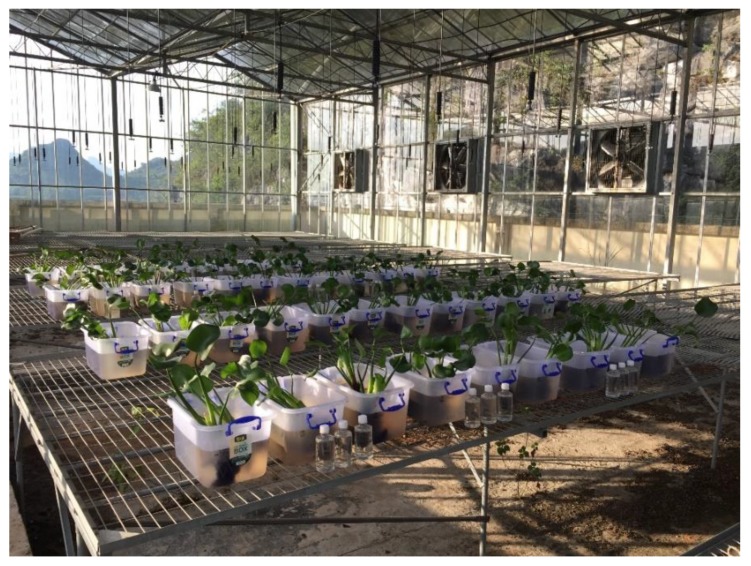
Experimental design.

All experiments were conducted in controllable greenhouses at the Huixian Karst Wetland Ecological Base (Guilin, Guangxi, China). Eight liters of experimental water and 150 g of similarly sized *E. crassipes* plants with a 1:3 root to stem/leaf mass ratio were placed into individual polyethylene vessels and cultivated at 25 ± 3 ℃ under natural light conditions for 24 days. The experiments were performed in triplicate with the inclusion of a control group. Deionized water was added to each experimental vessel regularly to compensate for water absorption and transpiration and keep the overall water volume constant. Figure 1 illustrates the experimental setup and Table 2 summarizes the experimental parameters. The initial Pb concentration in the experimental water was adjusted to 0.5 mg/L. The Pb concentration was adjusted by adding an appropriate amount of Pb(NO_3_)_2_ to the experimental water. The Ca^2+^ concentrations in karst and non-karst water were adjusted to 60, 80, and 100 mg/L, and 20 and 60 mg/L, respectively. Ca^2+^ concentrations were adjusted by adding appropriate amounts of analytical grade CaCl_2_ to the experimental water. The compounds were allowed to fully dissolve before being added to the experimental vessels.

Pb concentrations in water and elemental composition of *E. crassipes* were determined, and SEM and FTIR analyses of *E. crassipes* were conducted in this study. The Pb concentrations in water were tested every eight days. Polyethylene (PE) bottles were soaked in 10% HNO_3_ for 24 h and washed three times with both deionized and the experimental water before collecting the water samples. The water samples were filtered through a 0.45-µm micropore membrane filter, after which the filtrate was transferred to 50-mL PE bottles. HNO_3_ was then added to reduce the water pH to <2. The PE bottles were then sealed with paraffin wax and stored in a 4 °C refrigerator keeping the samples from direct light contact. The Pb concentrations in the water samples were determined via ICP-MS (iCAP Q, Thermo Fisher Scientific, Waltham, [MA], USA) according to the GB/T 5750.6-2006. The relative standard deviation (RSD) of the measured values of each index in the water sample was below 5.0% and the standard recovery was 80–120%. After the experimental treatment, *E. crassipes* was removed from the experimental vessel, rinsed with tap water, and allowed to dry, after which the roots, stems, and leaves were separated. The wet and dry weights of the samples were then measured. The elemental composition of roots, stems, and leaves were detected via ICP-MS according to the GB5009.268-2016. The detection limits of Ca, Mg, Na, K, P, Fe, Mn, Pb, Cd, Cu, and Zn were 1.00, 1.00, 1.00, 1.00, 1.00, 1.00, 0.10, 0.02, 0.002, 0.05, and 0.50 mg/kg, respectively. The fresh roots, stems, and leaves were freeze-dried via one-step tert-butanol freeze-drying. The dried samples were sprayed with gold and the morphology of the samples was assessed via SEM (JEM-6490 LV, JEOL) at a 20 kV accelerating voltage. The functional groups involved in the process of Pb absorption by *E. crassipes* were characterized via FTIR (Spectrum TWO, Perkin Elmer, Waltham, [MA], USA) using the KBr tablet method; 1.00 mg of sample and 200.00 mg of crushed KBr crystals were added to an agate mortar and fully ground. The mixtures were then transferred to a mold to prepare uniform and transparent ingots using a tablet press. Three ingots were prepared for each processed sample.

### 2.3. Data Processing

The experimental data were analyzed using the Origin 9 (OriginLab, Northampton, [MA], USA) Microsoft Excel 2010 (Microsoft, Redmond, [WA], USA) software, and the experimental values were expressed as the mean(s) ± standard deviation (SD) (*n* = 3) of three replicate experiments. The data normality was first verified; descriptive statistics and data exploration were performed using the SPSS 19 software (IBM, Armonk, [NY], USA). The data were considered to be normally distributed at a *p*-value ≥ 0.05. Afterward, variance homogeneity was verified, and mean comparisons, one-way ANOVA, and variance homogeneity were determined using SPSS 19. The variance was deemed homogeneous at a *p*-value > 0.05. One-way ANOVA coupled with the Tukey test was performed using SPSS 19 to identify significant differences between treatments. The statistical significance threshold was set at *p <* 0.05.

The results obtained in the experiments were used to determine the following parameters:

Removal rate (%): q = (C_0_-C)/C_0_×100%;

Bioconcentration amount (BCA): BC = CP-CP_0_;

Bioconcentration factor (BCF): BCF = CP/CS;

Translocation factor (TF): TF = CSL/CR,

Where C_0_ is the concentration of Pb in water before the experiment (mg/L); C is the concentration of Pb in water during the experiment (mg/L); CP is the Pb content in a given *E. crassipes* structure (mg/kg); CP_0_ is the initial Pb content in a given *E. crassipes* structure (mg/kg); CS is the concentration of Pb in water after the experiment (mg/kg); CSL is the content of Pb in stems and leaves (mg/kg); CR is the Pb content in roots (mg/kg).

## 3. Results and Discussion

### 3.1. Effect of Ca^2+^ Concentration on Pb Removal from Water

**Figure 2:**
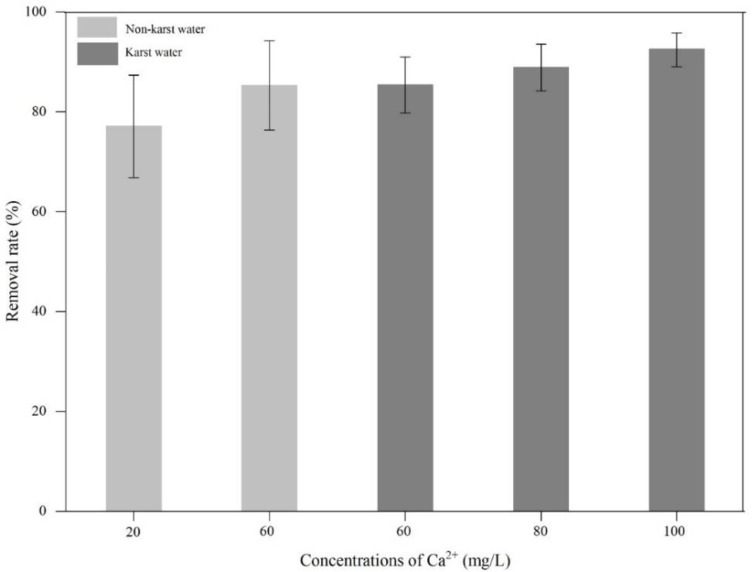
The removal of lead (Pb) in water by *Eichhornia crassipes* under different concentrations of calcium ion (Ca^2+^).

As shown in Figure 2, the *E. crassipes* Pb removal rate in water varied as a function of Ca^2+^ concentration. The Pb removal rate in karst water with 60 mg/L Ca^2+^ (85.31%) was higher than that of non-karst water with 20 mg/L Ca^2+^ (77.04%). The Pb removal rate was 85.31%, 88.87%, and 92.44% in karst water with 60, 80, and 100 mg/L Ca^2+^, respectively. In non-karst water with 20 and 60 mg/L Ca^2+^, the Pb removal rate was 77.04% and 85.24%, respectively. The significant difference between the Pb removal rate in water with different Ca^2+^ concentrations was tested using SPSS 19. The *p*-value between groups was 0.004 (i.e., < 0.01), indicating that the difference between the removal rates of Pb in water with different Ca^2+^ concentrations was very significant. This study found that the Pb removal rate increased with higher Ca^2+^ concentrations in both karst and non-karst water, and therefore we concluded that high Ca^2+^ concentrations facilitate Pb removal from water by *E. crassipes*. Calile et al. [16] demonstrated that an increased Ca^2+^ concentration could reduce the solubility of heavy metals in water. Yin et al. [20] analyzed the effect of liming (91% calcium carbonate) on Pb weathering in sand berms and found that the total Pb concentrations in the limed plots were lower than those in the plots without lime. Yang et al. [21] demonstrated that CaCl_2_ greatly enhanced the Pb removal rate from blast furnace dust, which was consistent with present results. Nonetheless, Pb removal rates at a 60 mg/L Ca^2+^ concentration were only slightly lower in non-karst water (85.24%) than in karst water (85.31%). It can be speculated that other hydrochemical differences (i.e., in addition to Ca^2+^) between karst and non-karst water may modulate Pb removal by *E. crassipes*, such as HCO_3_^−^ concentration, as proposed by Zhang et al. [19]. Therefore, the effects of HCO_3_^−^ on Pb removal from karst water by *E. crassipes* should be analyzed in further studies.

### 3.2. Effect of Ca^2+^ Concentration on Pb Absorption in E. crassipes

The effect of Ca^2+^ concentration on the elemental composition of different *E. crassipes* structures before and after Pb absorption were tested by ICP-MS (Table 3). The Pb bioconcentration amount (BCA), bioconcentration factor (BCF), and translocation factor (TF) were then calculated (Table 4). Significant differences between the Pb BCA in *E. crassipes* in water with different Ca^2+^ concentrations were identified with the SPSS 19 software. The *p*-value was 0.001 (< 0.01), indicating that the difference between the Pb BCAs was very significant. The Pb BCA in *E. crassipes* roots in karst water with 60 mg/L Ca^2+^ (1763 mg/kg) was lower than that in non-karst water with 20 mg/L Ca^2+^ (2143 mg/kg). The Pb BCA in *E. crassipes* roots in karst water with 60, 80, and 100 mg/L Ca^2+^ were 1763, 1317, and 1095 mg/kg, respectively. Furthermore, in non-karst water with 20 and 60 mg/L of Ca^2+^, the Pb BCA in *E. crassipes* roots was 2143 mg/kg and 1881 mg/kg, respectively. This study found that Pb BCA in *E. crassipes* roots decreased with increased Ca^2+^ concentrations in both karst and non-karst water. Therefore, Pb removal rates from water and BCA in roots of *E. crassipes* exhibited opposite trends in response to Ca^2+^ concentration. The statistical correlations between Pb BCA in *E. crassipes* and Pb removal rate from water as a function of Ca^2+^ concentration were tested via Spearman rank correlation analysis with SPSS 19. The correlation was significant at a 0.01 confidence level (bilateral). Xie [22] analyzed the effects of Ca^2+^ on the Pb stress response of *Epipremnum aureum* and reported that Ca^2+^ significantly decreased Pb enrichment in roots. Moreover, Shi et al. [23] reported that exogenous Ca^2+^ addition could significantly reduce heavy metal absorption by the roots of *Wedelia trilobata*. Li et al. [24] also demonstrated that Ca^2+^ addition significantly reduced Pb enrichment in *Lentinus edodes*, which was consistent with our results.

BCF is among the most important indicators of heavy metal absorption in plants. It is expressed as the ratio between the heavy metal concentrations in aquatic plant tissues to the concentration in water, whereby a BCF > 1 is indicative of heavy metal accumulation in plants [25]. The Pb BCF in *E. crassipes* roots in 60 mg/L Ca^2+^ karst water (30,306) was higher than that in non-karst water with 20 mg/L Ca^2+^ (21,583), which was consistent with the Pb removal rate in water. The highest Pb BCF (31,531) was observed in 80 mg/L Ca^2+^ karst water. The Pb BCF in *E. crassipes* roots in 60 mg/L Ca^2+^ non-karst water (35,186) was higher than that in 20 mg/L Ca^2+^ non-karst water.

TF represents the ability of plants to transfer the absorbed heavy metals from underground to aboveground structures. A TF value > 1 indicates that the plant has a strong ability to transport a certain heavy metal [26]. The Pb TF in *E. crassipes* in water with different Ca^2+^ concentrations was below 1, indicating that the Pb transport ability of *E. crassipes* was weak regardless of Ca^2+^ concentration. High Ca^2+^ concentrations in water had no significant effect on the ability of *E. crassipes* to transfer Pb from roots to stems and leaves. *E. crassipes* has the ability to protect leaves and stems from the toxic effects of Pb on photosynthesis. Plants commonly absorb harmful heavy metals into their roots as a defense mechanism to prevent toxicity to leaves and stems and to maintain photosynthesis and other metabolic activities [27,28]. The lowest Pb TF occurred in 80 mg/L Ca^2+^ karst water (0.0650). The Pb TF in *E. crassipes* in 60 mg/L Ca^2+^ non-karst water (0.211) was lower than that in non-karst water with 20 mg/L Ca^2+^ (0.256). A similar study reported that certain Ca^2+^ concentrations could inhibit the transport of heavy metals from underground to aboveground plant structures [15].

### 3.3. Morphology Analysis

**Figure 3:**
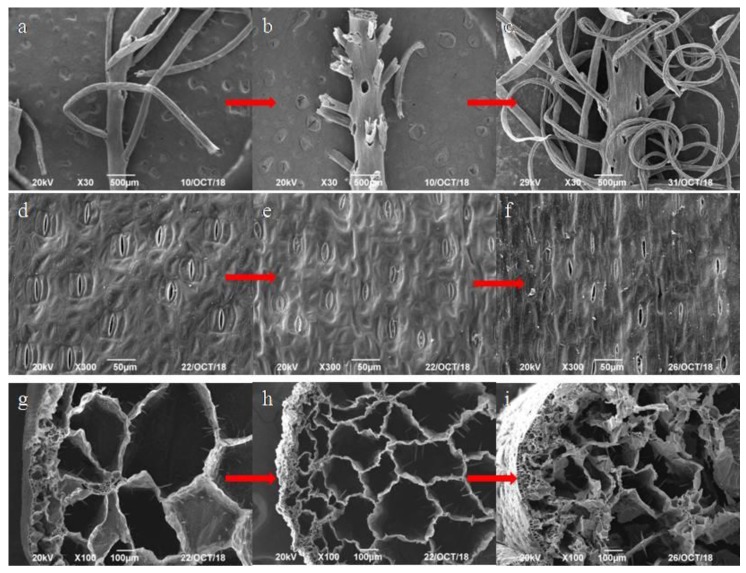
SEM images of (**a**) roots before experiment, (**b**) roots in karst water with 60 mg/L Ca^2+^, (**c**) roots in karst water with 80 mg/L Ca^2+^, (**d**) leaves before experiment, (**e**) leaves in karst water with 60 mg/L Ca^2+^, (**f**) leaves in karst water with 80 mg/L Ca^2+^,(**g**) stems before experiment, (**h**) stems in karst water with 60 mg/L Ca^2+^, (**i**) stems in karst water with 80 mg/L Ca^2+^.

The morphology of roots, stems, and leaves of *E. crassipes* in water was examined by SEM. The SEM images of roots, stems, and leaves of *E. crassipes* in 60 and 80 mg/L Ca^2+^ karst water are illustrated in Figure 3.

Roots are vital structures for plant growth, as they are mainly responsible for absorbing water and inorganic salts. The root system is comprised of taproots and fibrous roots, which are largely responsible for pollutant removal. Figure 3a shows that there are voids between the taproots and fibrous roots of *E. crassipes*. Many of these voids develop into air passages, which provide buoyancy, support, and favorable conditions for roots to absorb heavy metals and enhance the adaptability of *E. crassipes* to aquatic environments. The cell walls of roots act as a biological semipermeable membrane, which is difficult for large molecular weight substances, nonionic compounds, and colloids to penetrate. There is also a large contact area between the root system and the water surface, which forms a filter layer capable of absorbing a variety of heavy metals to improve water quality [8,29]. As shown in Figure 3b,c, the voids between the taproots and fibrous roots did not change significantly in karst water with 60 and 80 mg/L Ca^2+^. Moreover, fibrous roots were more fragile in karst water with 60 mg/L than in karst water with 80 mg/L Ca^2+^. This study demonstrated that Pb had low toxicity on *E. crassipes* roots, which was attributable to the high Ca^2+^ concentrations in karst water. Similar studies also showed that high concentrations of Ca^2+^ could reduce the toxicity of heavy metals to other plants [23,24].

Leaves are important plant structures that are responsible for photosynthesis, gas exchange, and transpiration. Leaves have a relatively large surface area and contain many stomas, distributed evenly on the epidermis. Figure 3d illustrates the highly developed stoma structure of *E. crassipes* leaves, which enhances the photosynthesis and transpiration capacity of *E. crassipes* and promotes a more efficient heavy metal absorption in water through the roots. As shown in Figure 3e, most stomas were closed after absorbing Pb in 60 mg/L Ca^2+^ karst water. A similar study reported that *E. crassipes* possesses many small stomas, which closed upon safranin exposure [30]. Heavy metals enter leaves through the open stomas and then compromise the physiological activities of plants [31]. Brunet et al. [32] demonstrated that heavy metals disrupted carbon sequestration, which was attributed to stomatal closure. As shown in Figure 3f, when the Ca^2+^ concentration was 80 mg/L in karst water, a few stomas were still open. Therefore, our results suggest that Pb seriously damaged leaf morphology and high Ca^2+^ concentrations in karst water alleviated this Pb-induced morphological damage.

Stems are comprised of a highly developed intercellular epidermis, spongy cortex, vascular bundles, and medulla; the vascular bundle is arranged as a ring around the medulla, forming a complex network. As shown in Figure 3g, these structures are comprised of many interconnected air chambers and different sized airways, which facilitate gas exchange and storage. As shown in Figure 3h, after absorbing Pb in 60 mg/L Ca^2+^ karst water, the cortex became atrophied and thin, and the volume of the annular vascular bundle structure decreased. As shown in Figure 3i, the cortical morphology of stems was less affected in 80 mg/L Ca^2+^ karst water. These results suggest that *E. crassipes* stems were seriously damaged by Pb; however, the high concentration of Ca^2+^ in karst water reduced the Pb-induced morphological effects in stems.

The voids in roots, leaf stomas, air chambers, and stem airways provided favorable conditions for *E. crassipes* to accumulate large amounts of Pb. The morphology of stems and leaves was damaged more seriously than that of roots after absorbing Pb, indicating that roots had a stronger tolerance to Pb than stems and leaves. The results of this study demonstrate that roots were the main *E. crassipes* structures responsible for Pb absorption. Moreover, increased Ca^2+^ concentrations in water led to a reduction in Pb-induced damage to roots, stems, and leaves, thereby enhancing the adaptability of *E. crassipes* to Pb stress.

### 3.4. Functional Group Analysis

**Figure 4:**
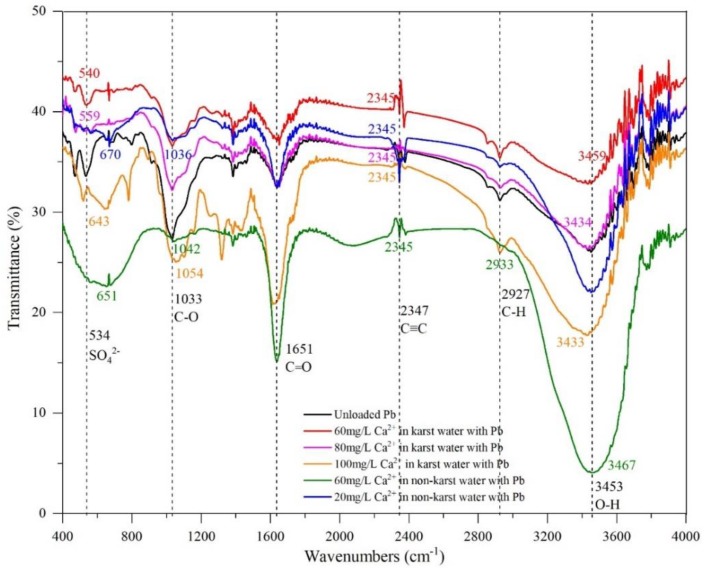
Transmittance spectrum of *E. crassipes* roots before and after absorbing Pb in water with different Ca^2+^ concentrations.

Figure 4 illustrates the transmittance spectrum of *E. crassipes* roots before and after absorbing Pb in water with different Ca^2+^ concentrations. FTIR spectra can provide a wealth of useful information on functional groups [33]. As shown in Figure 4, the FTIR spectrum of *E. crassipes* roots exhibited a few intense bands associated with organic functional groups. The broad band observed at 3453 cm^−1^ was attributed to O-H stretching vibration. The spectra peak at 2927 cm^−1^ represented -CH_3_ stretching vibration. The spectrum band observed at 2347 cm^−1^ reflected C≡C stretching vibration. The spectra peak at 1651 cm^−1^ represented protein C=O stretching vibration. The spectra band observed at 1033 cm^−1^ reflected C-O stretching vibration. The spectra peak at 534 cm^−1^ was attributed to SO_4_^2−^ stretching vibration [34].

As shown in Figure 4, both C≡C peaks shifted from 2347 to 2345 cm^−1^, and the peak strength of C≡C in karst water with 60 mg/L Ca^2+^ was smaller than that in non-karst water with 20 mg/L Ca^2+^. The protein C=O peak on the cell walls at 1651cm^−1^ did not shift, but the peak strength in karst water was greater than that in non-karst water, indicating that the cell wall structure changed when the protein C=O interacted with Pb [35]. The SO_4_^2−^ peak at 534 cm^−1^ shifted to 540 cm^−1^ in karst water but shifted to 670 cm^−1^ in non-karst water. The peak strength of the SO_4_^2−^ stretching vibration in karst water was greater than that in non-karst water. Therefore, this study demonstrated that C≡C, protein C=O, and SO_4_^2−^ played different roles in karst and non-karst water. O-H only had an effect in karst water, and the O-H peak shifted from 3453 to 3459 cm^−1^. The C-O peak only reacted in non-karst water, where it shifted from 1033 to 1036 cm^−1^. It is speculated that the different hydrochemical characteristics of karst and non-karst water may lead to the observed differences in functional groups, spectrum shapes, and the displacement and strength of some peaks, which should be examined in future research.

No significant changes in FTIR spectra shape were observed in karst water with 60, 80, and 100 mg/L Ca^2+^, suggesting that high Ca^2+^ concentrations in karst water did not obviously change the basic chemical composition of roots. Li [36] reported similar results in *E. crassipes* exposed to heavy metals in polluted aquatic environments. However, the displacement and strength of some peaks changed. The O-H peaks at 3453 cm^−1^ shifted to 3459, 3434, and 3433 cm^−1^ in karst water with 60, 80, and 100 mg/L Ca^2+^, respectively. Moreover, the O-H peak strength increased with increased Ca^2+^ concentrations, indicating that Ca^2+^ concentrations in karst water increased the occurrence of hydroxyl hydrogen bonds on the surface of *E. crassipes*. The C≡C peak at 2347 cm^−1^ had a same displacement, but the peak strength decreased with increased Ca^2+^ concentrations, suggesting that the strength of alkynyl groups on the surface of *E. crassipes* weakened as a result of combining Pb with the high Ca^2+^ concentrations in karst water. Acetylene compounds are easily oxidized; however, higher Ca^2+^ concentrations in karst water reduced the oxidization of the acetylene bond of alkynyl. The protein C=O peak at 1651 cm^−1^ did not shift, but the peak strength increased with higher Ca^2+^ concentrations, suggesting that cell wall structure changed when exposed to Pb. This result was consistent with a previous study that compared infrared spectroscopy between endogenous and exogenous metals in phytoplankton [37]. Protein C=O bonds played a more important role during Pb exposure. Cells absorb heavy metal ions through extracellular space, and the cell walls of plants are the first barrier against pollutants. The polyuronic acid and cellulose in cell walls provide a large number of exchange sites for pollutants [38]. Pb has a notably large ion radius and weak coordination ability. Therefore, it is difficult for Pb to enter into the cytoplasm through the cell wall and plasma membrane. *E. crassipes* absorbs Pb mainly through cell walls, as well as through non-metabolic interstitial diffusion between cells. Pb begins to enter the cytoplasm through the cell walls and plasma membranes after reaching saturation, and the toxicity of Pb to *E. crassipes* is low when the absorption speed is fast [39]. The peak strength of SO_4_^2−^ at 534 cm^−1^ shifted to 540, 559, and 643 cm^−1^ in karst water with 60, 80, and 100 mg/L Ca^2+^, respectively. The increased SO_4_^2−^ displacement indicated the presence of a small amount of sulfuric acid in *E. crassipes*. SO_4_^2−^ played a more significant role with increased Ca^2+^ concentrations. This study showed that when the Ca^2+^ concentration increased from 60 to 80 mg/L, O-H, C≡C, protein C=O, and SO_4_^2−^ responded to Pb exposure. When the Ca^2+^ concentration increased to 100 mg/L, a new functional group (C-O) at 1033 cm^−1^ was identified. The occurrence of O-H at 3453 cm^−1^ and C-O at 1033 cm^−1^ proved that *E. crassipes* contains alcoholic hydroxyl groups. Alcohol compounds are easily oxidized [34]. In this study, the results showed that alcoholic hydroxyls played a more significant role in karst water with high Ca^2+^ concentrations.

Our results suggest that high Ca^2+^ concentrations play an important role in regulating the response of functional groups in *E. crassipes* during Pb exposure. Some organic compounds such as alcohol and sulfate resins are known to be present in *E. crassipes* and both alcoholic hydroxyls and SO_4_^2−^ play a more significant role in water with high Ca^2+^ concentrations. Increases in Ca^2+^ concentrations in water reduce the oxidization of alkynyl acetylene bonds and therefore protect the cell of *E. crassipes* form heavy metal-induced damage.

### 3.5. Ion Exchange Analysis

**Figure 5:**
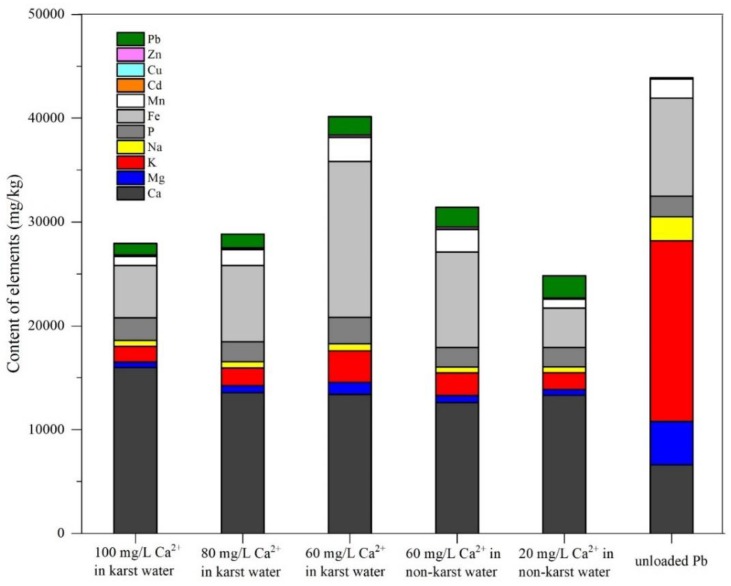
Elemental composition and distribution in *E. crassipes* roots before and after Pb absorption from water with different Ca^2+^ concentrations.

Figure 5 illustrates the elemental composition and distribution in *E. crassipes* roots before and after Pb absorption from water with different Ca^2+^ concentrations. As shown in Figure 5, the Pb and Ca content in *E. crassipes* roots increased after absorbing the waterborne Pb. In contrast, the Mg, Na, and K contents in *E. crassipes* roots decreased. These observations are suggestive of cation exchange, whereby Pb was exchanged for Mg, Na, and K during the process of Pb absorption form water. Li et al. [34] demonstrated that Ca, Mg, and K were depleted from *E. crassipes* roots, while Cu and Cr were accumulated upon Pb exposure. Xia [40] also demonstrated that Pb was exchanged for K, Ca, and Mg during the absorption of Pb by *E. crassipes*, which was consistent with our results. The absorption of Ca and the depletion of Mg, Na, and K was more pronounced in *E. crassipes* roots when the Ca^2+^ concentrations in karst water were higher, indicating that high Ca^2+^ concentrations stimulated cation exchange. Furthermore, Na: K proportions in *E. crassipes* roots (0.228, 0.341, and 0.362) and Ca: Mg proportions (11.6, 19.8, and 29.8) increased in roots with increased Ca^2+^ concentrations in karst water (60, 80, and 100 mg/L).

### 3.6. Elemental Biogeochemical Behavior

Plants are selective in absorbing elements. Generally, plants absorb elements that are richer, easier to obtain, and that can serve a particular function. Ca is an essential element for plants. It is also an important plant growth regulator and mediates the response to environmental changes [11]. Pb is a nonessential trace element that occurs in nature; however, its accumulation can lead to serious toxic effects. Pb accumulation in plants mainly affects photosynthesis and transpiration. In our study, higher Ca^2+^ concentrations in the water led to decreased Pb BCA and higher Ca absorption in *E. crassipes* roots (Table 3). These results suggest that there was a competitive relationship between Ca and Pb in the process of being absorbed by *E. crassipes*. High Ca^2+^ concentrations in water therefore inhibited the absorption of Pb.

Pb concentration and form in natural water are affected by the concentrations of CO_3_^2−^, SO_4_^2−^, and OH^−^, which facilitate the precipitation of Pb into PbCO_3_, PbSO_4_, and Pb(OH)_2_. The concentration of Pb in water is controlled by hydroxide ions, and the main complexation reactions in natural waters can be described as follows:

(1) $$Pb^{2+}+OH^{-}=PbOH^{+}$$

(2) $$Pb^{2+}+2OH^{-}=Pb{\left(OH\right)}_{2}^{0}$$

(3) $$Pb^{2+}+3OH^{-}=Pb{\left(OH\right)}_{3}^{-}$$

(4) $$Pb^{2+}+Cl^{-}=PbCl^{+}$$

(5) $$Pb^{2+}+2Cl^{-}=Pb{\left(Cl\right)}_{2}^{0}$$

When the pH of water is 8.5, the proportions of Pb complexes are typically 88% PbOH^+^, 10% PbCO_3_, and 2% (PbCl^+^ + PbSO_4_) [41]. OH^−^ and CO_3_^2−^ can form ion pairs with Ca^2+^, but this mainly occurs in strongly alkaline solutions (pH > 9.5). The pH of karst water used in our experiments was 7.25 and pH varied between 8.27 and 8.60 as the Ca^2+^ concentration increased. The pH of natural water mainly depends on its free carbon dioxide (CO_2_) content and carbonate equilibrium. *E. crassipes* absorbs a substantial amount of CO_2_ during photosynthesis and pH likely increased due to photosynthesis-linked CO_2_ uptake. OH^−^ tended to form complexes with Pb rather than with Ca^2+^ due to the increase of pH in water. Some of the dissolved Pb existed in the form of PbOH^+^, PbCO_3_, PbCl^+^, and PbSO_4_. However, the solubility and mobility of Pb decreased because PbCO_3_ and PbSO_4_ are insoluble in water, which affected the absorption of Pb by *E. crassipes*. As the pH of the water increased with the increase of Ca^2+^ concentrations, PbCO_3_ and PbSO_4_ became more abundant.

As a result of increased Ca^2+^ concentrations, the precipitation of PbCO_3_ and PbSO_4_ and the competitive relationship between Ca and Pb in water led to a decrease in Pb BCA in *E. crassipes* and a higher Pb removal rate from water. Moreover, high Ca^2+^ concentrations alleviated the Pb-induced morphological damage to roots, stems, and leaves.

## 4. Conclusions

In summary, high Ca^2+^ concentrations improved the Pb removal efficiency of *E. crassipes* in karst water, but reduced the Pb BCA in *E. crassipes* roots. In contrast, the Ca BCA in roots increased, with increased Ca^2+^ concentrations in karst water. The highest bioconcentration factor and the lowest transport factor of Pb occurred in 80 mg/L Ca^2+^ karst water. The differences in bioconcentration amounts and removal rates induced by higher Ca^2+^ concentrations were attributed to Pb precipitation (i.e., as PbCO_3_ and PbSO_4_) and the competitive relationship between Ca and Pb in karst water. High Ca^2+^ concentrations in karst water increased the strength of cation exchange, whereby Pb was exchanged for Mg, Na, and K. Moreover, high Ca^2+^ concentrations in karst water alleviated Pb-induced structural damage to roots, stems, and leaves, and protected the cell wall of *E. crassipes*. Ca^2+^ also regulated the functional groups involved during Pb exposure in *E. crassipes*. The oxidization of alkynyl acetylene bonds decreased, and alcohol compounds and sulfate resin were both important during Pb pressing in water with high Ca^2+^ concentrations. Therefore, increasing the supply of Ca^2+^ could reduce the uptake of Pb and alleviate Pb toxicity in *E. crassipes*.

## Figures and Tables

**Table 1:** Hydrochemical parameters of karst water and non-karst water used in the experiments.

Parameters	Karst Water	Non-karst Water	Parameters	Karst Water	Non-karst Water
pH	7.25	7.50	K^+^/mg·L^−1^	3.46	1.95
Pb/µg·L^−1^	<0.07	<0.07	Na^+^/mg·L^−1^	1.02	1.16
Al/µg·L^−1^	38.5	41.9	Ca^2+^/mg·L^−1^	57.06	23.42
Zn/µg·L^−1^	0.88	4.29	Mg^2+^/mg·L^−1^	3.66	1.33
Cr/µg·L^−1^	4.52	1.30	NH_4_^+^/mg·L^−1^	0.060	<0.02
Ni/µg·L^−1^	1.40	0.91	Cl^−^/mg·L^−1^	3.04	5.25
Co/µg·L^−1^	<0.03	<0.03	SO_4_^2−^/mg·L^−1^	9.32	9.00
Cd/µg·L^−1^	<0.06	<0.06	HCO_3_^−^/mg·L^−1^	173.56	58.61
Mn/µg·L^−1^	44.6	6.92	CO_3_^2−^/mg·L^−1^	0.00	0.00
As/µg·L^−1^	1.16	0.64	NO_3_^−^/mg·L^−1^	0.84	8.17
Hg/µg·L^−1^	37.8	1.15	NO_2_^−^/mg·L^−1^	<0.002	0.002
Cu/µg·L^−1^	0.48	0.98	F^−^/mg·L^−1^	0.078	0.080
CO_2_/mg·L^−1^	8.95	3.83	PO_4_^3−^/mg·L^−1^	0.23	0.040
SiO_2_/mg·L^−1^	5.35	5.25	OH^−^/mg·L^−1^	0.00	0.00
Permanganate index (COD_Mn_) /mg·L^−1^	2.07	<0.5	Total iron (TFe) /mg·L^−1^	0.13	0.012

**Table 2:** Experimental parameters of the effects of calcium ion (Ca^2+^) concentrations on *Eichhornia crassipes* absorbing lead (Pb) in water.

Experimental Water	Heavy Metal	Compounds	Initial concentration of Pb (mg/L)	Volume of Water (L)	Mass of *Eichhornia crassipes* (g)	Mass of Pb(NO_3_)_2_ (g)	Concentration of Ca^2+^ in Water (mg/L)	Mass of CaCl_2_ (g)
Karst Water	Pb	Pb(NO_3_)_2_	0.5	8	150	0.0064	60	0
							80	0.4430
							100	0.8861
Non-karst Water	Pb	Pb(NO_3_)_2_	0.5	8	150	0.0064	20	0
							60	0.8861

**Table 3:** Elemental composition of different *E. crassipes* structures before and after Pb absorption in water with different Ca^2+^ concentrations (mg/kg).

Water Body	Concentrations of Ca^2+^ in Water (mg/L)	Parts	Ca	Mg	K	Na	P	Fe	Mn	Pb	Cd	Cu	Zn
		Roots	13,401	1157	3021	690	2529	15,036	2320	1771	0.448	14.8	212
Karst Water	60	Stems and Leaves	17,800	1251	15,301	663	2932	2895	302	177	0.0933	177	77.8
		Whole Plant	16,700	1228	12,231	690	2831	5930	806	576	0.182	136	111
		Roots	13,555	684	1703	581	1941	7351	1537	1324	0.520	9.47	155
	80	Stems and Leaves	20,600	1294	10,938	612	3287	491	225	86.5	0.134	31.4	45.0
		Whole Plant	18,839	1142	8629	604	2950	2206	553	396	0.230	25.9	72.5
		Roots	15,968	537	1535	555	2171	5047	889	1103	0.247	8.72	119
	100	Stems and Leaves	22,100	905	6181	610	3196	770	300	173	0.109	37.2	52.7
		Whole Plant	20,567	813	5020	596	2940	1839	447	406	0.144	30.1	69.3
Non-karst Water		Roots	12,610	671	2173	574	1903	9183	2180	1888	0.331	13.3	220
	60	Stems and Leaves	20,900	1086	13,391	651	3250	964	361	398	0.133	38.2	57.4
		Whole Plant	18,828	982	10,586	632	2913	3019	816	770	0.182	32.0	98.0
		Roots	13,316	576	1608	557	1859	3801	838	2151	0.204	9.70	108
	20	Stems and Leaves	17,600	1217	14,594	610	2809	574	308	550	0.0860	32.3	34.3
		Whole Plant	16,529	1057	11,348	597	2572	1381	440	950	0.116	26.6	52.7
		Roots	6600	4200	17,400	2300	2000	9442	1817	7.67	0.360	9.25	98.1
Unloaded		Stems and Leaves	15,500	1700	32,800	830	2900	279	192	0.730	0.0370	18.9	38.7
		Whole Plant	13,275	2325	28,950	1198	2675	2570	598	2.46	0.118	16.5	53.6

**Table 4:** Enrichment of Pb in different *E. crassipes* structures in water with different Ca^2+^ concentrations.

Water Body	Concentrations of Ca^2+^ in Water (mg/L)	Concentration of Pb in Water After the Experiment (mg/kg)	Removal Rate (%)	Parts	BCA (mg/kg)	BCF	TF
Karst Water	60			Roots	1763	30,360	\
		0.058 ± 0.018	85.31 ± 5.61	Stems and Leaves	177	3039	\
				Whole Plant	576	9866	0.100
	80			Roots	1317	31,531	\
		0.042 ± 0.013	88.87 ± 4.68	Stems and Leaves	85.7	2059	\
				Whole Plant	396	9426	0.0650
	100			Roots	1095	28,761	\
		0.038 ± 0.015	92.44 ± 3.40	Stems and Leaves	173	4523	\
				Whole Plant	406	10,578	0.157
Non-karst Water	60			Roots	1881	35,186	\
		0.054 ± 0.019	85.24 ± 8.91	Stems and Leaves	398	7424	\
				Whole Plant	770	14,357	0.211
	20			Roots	2143	21,583	\
		0.010 ± 0.041	77.04 ± 10.26	Stems and Leaves	549	5517	\
				Whole Plant	950	9534	0.256
